# The In Vitro Bioactivity, Degradation, and Cytotoxicity of Polymer-Derived Wollastonite-Diopside Glass-Ceramics

**DOI:** 10.3390/ma10040425

**Published:** 2017-04-18

**Authors:** Amanda De Castro Juraski, Andrea Cecilia Dorion Rodas, Hamada Elsayed, Enrico Bernardo, Viviane Oliveira Soares, Juliana Daguano

**Affiliations:** 1 Centro de Engenharia, Modelagem e Ciências Sociais Aplicadas, Federal University of ABC, Santo André 09210-580, Brazil; amanda.juraski@gmail.com (A.D.C.J.); andreadorion@gmail.com (A.C.D.R.); 2 Dipartimento di Ingegneria Industriale, University of Padova, Padova 35131, Italy; elsisy_chem@yahoo.com; 3 Ceramics Department, National Research Centre, El-Bohous Street, 12622 Cairo, Egypt; 4 Departamento de Ciências, State University of Maringá, Maringá 87360-000, Brazil; soares.v.o@gmail.com

**Keywords:** glass-ceramics, polymer-derived ceramics, bioactivity, cytotoxicity

## Abstract

Ca-Mg silicates are receiving a growing interest in the field of bioceramics. In a previous study, wollastonite-diopside (WD) glass-ceramics were successfully prepared by a new processing route, consisting of the heat treatment of a silicone resin embedding reactive oxide particles and a Ca/Mg-rich glass. The in vitro degradation, bioactivity, and cell response of these new WD glass-ceramics, fired at 900–1100 °C for 1 h, as a function of the Ca/Mg-rich glass content, are the aim of this investigation The results showed that WD glass-ceramics from formulations comprising different glass contents (70–100% at 900 °C, 30% at 1100 °C) exhibit the formation of an apatite-like layer on their surface after immersion in SBF for seven days, thus confirming their surface bioactivity. The XRD results showed that these samples crystallized, mainly forming wollastonite (CaSiO_3_) and diopside (CaMgSi_2_O_6_), but combeite (Na_2_Ca_2_Si_3_O_9_) crystalline phase was also detected. Besides in vitro bioactivity, cytotoxicity and osteoblast adhesion and proliferation tests were applied after all characterizations, and the formulation comprising 70% glass was demonstrated to be promising for further in vivo studies.

## 1. Introduction

Ceramics based on a CaO-MgO-SiO_2_ system have been widely investigated because of their bioactivity [1], controlled biodegradation rate [2], and biocompatibility [3]. These materials are quite versatile, because they can be used for medical and dental applications: as powders [4], dense ceramics [5], composites [6,7], and scaffolds [8].

Some of the CaO-MgO-SiO_2_ bioceramics are designed to form both wollastonite (CaSiO_3_) and diopside (CaMg(SiO_3_)_2_), considering the properties of these two crystalline phases [9]. In simulated body fluid (SBF), wollastonite is significantly more reactive than diopside. The different solubility of these phases controls the microstructure developed in the SBF solution [10]: wollastonite controls the formation of the hydroxyapatite (HA) layer on the surface of the bioceramic, whereas the mechanical stability of the wollastonite-diopside bioceramic is often attributed to diopside [11]. Another interesting phase that may form in Na-containing systems is combeite (Na_2_Ca_2_Si_3_O_9_), which is also known to develop from the crystallization of Bioglass^®^ [12]. Although this crystal phase can decrease the apatite formation kinetics on the Bioglass^®^ surface, it has been considered in the design of scaffolds because of its good resorbability and mechanical properties [13,14,15].

Many synthesis routes can be used to obtain bioceramics, according to the desired properties, morphology, and microstructure [16]. Silicone polymeric precursors, coupled with reactive oxide fillers and glass particles, offer a promising alternative route. The processing of preceramic polymers as a route to obtain advanced ceramics has been extensively studied in the last 40 years [17]; the extension to bone tissue engineering applications is far more recent [18,19,20].

The use of polymeric precursors offers the fundamental advantages of combining the synthesis and shaping of ceramics with complex geometries. Nevertheless, the control of shrinkage and porosity formation during the pyrolysis step remain problematic [21]. In some cases, the shrinkage may approach 50 vol %, and cracks and pores due to the ceramic conversion (owing to the release of gaseous by-products) may compromise the integrity of the material and its application [22]. Cracks may be significantly reduced when operating with fillers of a various nature [19]. The porosity, on the other hand, is often desirable, especially when applying polymer-derived ceramics as scaffolds for bone tissue engineering; in addition, preceramic polymers can be easily foamed [23,24]. Glass particles may be considered as a particular kind of reactive filler: as an example, biocompatible and complex-shaped porous scaffolds were successfully fabricated by powder-based 3D-printing, using mixtures of a preceramic polymer, CaCO_3_ (providing CaO), and glass powder, leading to apatite–wollastonite glass-ceramics. The crystallization was found to depend on both the devitrification of glass and silicone-oxide interactions [25].

Previous research indicated that the crystal phases resulting from Ca/Mg-rich glass crystallization and silicone-oxide filler interactions may coincide. Wollastonite–diopside (WD) glass-ceramic foams were developed from the thermal treatment of silicone polymers filled with CaO/MgO precursors and Ca/Mg-rich glass powders [26,27]. The foaming was achieved by water vapor release, below 350 °C, in a silicone still in the state of a liquid polymer, associated with the decomposition of borax (Na_2_B_4_O_7_·10H_2_O) or sodium phosphate dibasic heptahydrate (Na_2_HPO_4_·7H_2_O) as “foaming fillers”. The cellular structure could be “freezed”, owing to the simultaneous cross-linking of the silicone. Both borate and phosphate led to liquid phase formation upon firing (in air), promoting ionic interdiffusion and the formation of silicate phases, but they were unable to offer a sufficient relaxation of the stresses caused by the thermal transformation of the polymeric matrix and crystallization. The use of Ca/Mg-rich glass powders led to crack-free samples, owing to the enhanced viscous flow during firing, with significant improvements in the compressive strength. The Ca/Mg-rich glass addition did not significantly modify the crystalline phases of the glass-ceramic, since the glass crystallization during firing resulted in the same crystalline phases (wollastonite and diopside), formed by interactions between the silicone and CaO/MgO precursors [26].

The biocompatibility of WD glass-ceramics, from a liquid preceramic polymer (H62C—A liquid silicone from Wacker Chemie GmbH, Munich, Germany) and reactive fillers, with or without the addition of glass powders, has already been shown for foaming operated by the decomposition of sodium phosphate dibasic heptahydrate (Na_2_HPO_4_·7H_2_O), by Fiocco et al. [27]. The foams, in particular, exhibited positive results in terms of cell viability, according to the MTT (colorimetric assay based on (3-(4,5-dimethylthiazol-2-yl)-5-(3-carboxymethoxyphenyl)-2-(4-sulfophenyl)-2H-tetrazolium)) assay and LDH (Lactate dehydrogenase) activity tests. Analogous foams, obtained using borax (Na_2_B_4_O_7_·10H_2_O), have recently been subjected to extensive biological characterization, resulting in a confirmation of their biocompatibility and bioactivity [28]. However, nothing definitive can be said concerning the use of alternative polymers, which are solid at room temperature (such as MK—another commercial silicone, still from Wacker Chemie GmbH).

Solid polymers are normally mixed with the reactive fillers after being dissolved in isopropyl alcohol; the cross-linking upon drying impedes any foaming by water vapor release, but cellular structures may be achieved by different methods, e.g., by the application of direct or indirect 3D-printing. In fact, recent experiences have shown the feasibility of reticulated scaffolds by the direct ink writing (also known as “robocasting”, i.e., direct 3D-printing) of silicone-based pastes, as well as by powder 3D-printing (ink jet writing) on pure silicone or silicone-fillers [25,29,30,31].

The aim of the present work is to elucidate the biological characterization of WD glass-ceramics, obtained by silicone-based mixtures and different amounts of Ca/Mg-rich glass powders as an additional filler. The addition of glass powders, reacting with the ceramic residue left by the polymer upon different heat treatments, allows for the synthesis of glass-ceramics with different crystallized fractions. This process will be further used to design new scaffolds by 3D-printing.

## 2. Materials and Methods

### 2.1. Starting Materials

A commercially available silicone, MK (Wacker Chemie GmbH), was considered as a silica precursor, with a yield of 84 wt % [26]. The CaO and MgO precursors were CaCO_3_ (Sigma Aldrich, Gillingham, UK) and Mg(OH)_2_ (Industrie Bitossi, Vinci, Florence, Italy), respectively, with a particle size diameter under 10 μm. These precursors were added in amounts corresponding to a molar balance of CaO/MgO/SiO_2_ equal to 2/1/3, in turn corresponding to 50 mol % wollastonite and 50 mol % diopside. Sodium hydrogen phosphate (Na_2_HPO_4_, Sigma Aldrich, Gillingham, UK) was used as an additional filler, in a very limited amount (corresponding to 0.5 g of anhydrous Na_2_O·P_2_O_5_ for 10 g of the wollastonite-diopside mixture). Finally, a powdered Ca/Mg rich silicate glass with a particle size <60 μm (mean diameter ~5 μm), later referred to as G20CaII glass, was added in different amounts [26]. The chemical composition of the G20CaII glass is shown in Table 1.

### 2.2. Preparation of Glass-Ceramics

MK was dissolved in isopropanol (15 mL for 10 g of final glass-ceramic) and was then mixed with micro-sized fillers, including sodium hydrogen phosphate. The mixing was performed under magnetic stirring, followed by sonication for 10 min, which allowed a stable and homogeneous dispersion. The mixture (WD1 series) was poured into glass containers and dried at 80 °C overnight. Mixtures containing G20CaII glass (WD2 and WD3 series) were prepared in the same conditions. After drying, the silicone-based mixtures were in the form of solid fragments, which were converted into fine powders by ball milling at 350 rpm for 30 min. The powders were cold-pressed in a cylindrical steel die, applying a pressure of 20 MPa for 1 min, without using any binder. The samples were fired at 900 or 1100 °C for 1 h, using a heating rate of 1°/min. Glass-ceramics of 0.5 g, 16.6 mm in diameter, and approximately 1.7 mm in thickness, were obtained. For comparative purposes, (silicone-free) glass-ceramics from G20CaII glass powders were also prepared (WD4 series).

### 2.3. Microstructural Analysis

X-ray diffraction analysis (XRD) was used to investigate the development of crystalline phases upon heat treatment. XRD data were collected on powdered samples by means of a diffractometer (Bruker AXS, D8 Advance, Karlsruhe, Germany) using CuKα radiation. The samples were scanned at scattering angles, 2θ, from 10° to 60°, with a step size of 0.02° and a collection time of 2 s. The crystalline phases were identified using Joint Committee for Powder Diffraction Studies (JCPDS) standard diffraction patterns. The amount of the crystal phases (crystallized volume fraction) was determined according to the procedure used by Daguano et al. [32]. The Crystallinity Index, *CI*%, was calculated operating on the diffractogram of the glass-ceramics, by evaluating the crystalline area, *A*_C_, and the total area, *A*_T_ (*A*_T_ = amorphous + crystalline), combined in the Equation (1):

*CI*% = (*A*_C_/*A*_T_) × 100% (1)

Data management and analysis was performed using the software Origin 8.5.

To analyze the morphological structure, glass-ceramics samples were sputtered with gold and finally subjected to scanning electron microscopy (SEM, Quanta 250 SEM-FEI, FEI/Thermo Fisher Scientific, OR, USA).

### 2.4. In Vitro Bioactivity Test

The apatite forming ability of the synthesized materials was investigated according to the method described by Kokubo and Takadama [33]. The simulated body fluid (SBF) used in this study was cellular and protein-free, with a pH of 7.25. The volume of SBF used in the bioactivity tests is related to the surface area of the sample. According to the procedures described by ISO 23317-07 [34], for a dense material, the appropriate volume of solution should obey the following relationship:

*S*_a_/*V*_s_ = 0.1 cm**^−^**^1^ (2)

where *V*_s_ represents the volume of SBF (mL) and *S*_a_ represents the total geometric surface area of the sample (mm^2^).

The samples were soaked in the SBF solution for seven and 14 days. After the test time required for each sample, they were immersed in acetone for 10 s to remove the SBF and stop the surface reactions. After drying, both sample surfaces were analyzed to check for the formation of a hydroxycarbonate apatite (HCA) layer on the surface. The test was conducted in triplicate (*n* = 3).

The monitoring of surface changes in the samples after the in vitro bioactivity tests was performed by Fourier Transform Infrared Spectroscopy (FT-IR) using a spectrometer VARIAN 660-IR model (Agilent Technologies, Santa Clara, CA, USA) operating in reflectance mode with a 4 cm**^−^**^1^ resolution in the 4000–400 cm**^−^**^1^ region.

The apatite forming ability on glass-ceramics was followed by SEM (Quanta 250 SEM-FEI) and XRD analysis (Bruker AXS, D8 Focus), in the range 2θ = 20°–50°. The samples were coated with a thin gold layer before SEM analysis.

### 2.5. In Vitro Degradation Test

Disc samples (*n* = 10) were first weighed and then geometrically measured, by means of a precision caliper, in order to estimate the surface area. They were immersed in the Tris-HCl solution (pH 7.4, 37 °C) at a surface area and solution volume ratio of 0.1 cm^−1^. The solutions were renewed after 1, 3, 7, 14, and 21 days, respectively. The samples were taken out at scheduled time points, rinsed with deionized water three times, dried, and weighed. The weight loss (*W*_L_) was calculated as follows:

*W*_L_ = (*W*_0_ − *W*_d_)/*W*_0_ × 100% (3)

where *W*_0_ denotes the initial weight of the samples and *W*_d_ represents the weight of the dried samples after the scheduled immersion time. The pH of the medium was recorded after each immersion period at 37 °C.

### 2.6. In Vitro Cytotoxicity

Balb/c cells (ATCC, Manassas, VA, USA) were employed for the cytotoxicity analysis. Cells were maintained on a regular feeding regime in a cell culture incubator at 37 °C/5% CO_2_/95% air atmosphere. Cells were seeded into 96 well plates at a density of 2 × 10^4^ cells per well plate and incubated for 24 h, prior to testing with liquid extracts. The culture media used was DMEM (Thermo-Fischer, Carlsbad, CA, USA) supplemented with 10% fetal bovine serum (Atena Biotecnologica, Campinas, Brazil) and 1% antibiotic/antimycotic solution (Thermo-Fischer, Carlsbad, CA, USA).

The extracts were prepared using 12 samples of each group that were immersed in cellular medium (concentration of 6 cm^2^/1 mL—Superficial area per volume of supplemented culture medium) for three days. The cytotoxicity of the liquid extracts was evaluated using the Methyl Tetrazolium (MTS) assay in 96 well plates (Promega, Madison, WI, USA). Liquid extracts were added into wells containing Balb/c cells in culture medium. Each of the prepared plates was incubated for 24 h at 37 °C/5% CO_2_. The MTS assay was then added and the cultures were reincubated for a further 2 h (37 °C/5% CO_2_). Next, the cultures were removed from the incubator and the absorbance was measured at a wavelength of 490 nm (Molecular Devices ELISA reader, St. Louis, MO, USA). Aliquots of sterile media were used as a control, and cells were assumed to have metabolic activities of 100%. The cell viability was calculated as follows:

Cell viability = (*OD*_sample_/*OD*_control_) × 100% (4)

where *OD*_sample_ is the optical density of the sample and *OD*_control_ is the optical density of the control.

### 2.7. Cell Adhesion and Proliferation

MG63 cells (human osteosarcoma ATCC, Manassas, VA, USA) were cultured in Minimum Essential Medium (MEM) (Thermo-Fischer, Carlsbad, CA, USA) supplemented with 10% fetal bovine serum (Atena Biotecnologica, Campinas, Brazil) and 1% antibiotic/antimycotic solution (Thermo-Fischer, Carlsbad, CA, USA) in a cell culture incubator at 37 °C/5%CO_2_/95% air atmosphere.

As for the evaluations of cell adhesion and proliferation, cells were seeded onto the disc sample surfaces in 24 well polystyrene plates, at a cell density of 2 × 10^4^ cells/well, and incubated at 37 °C in a humidified atmosphere with 5% CO_2_. After 3, 7, 14, 21, and 27 days, cultures were fixed with 4% formaldehyde in 0.9% sodium chloride solution for 30 min. The samples were then washed with the same solution, dehydrated in a graded series of alcohol, and stained with 2% Alizarin red (Sigma Aldrich, St. Louis, MO, USA) (pH 4.2). Finally, they were photographed using a confocal laser microscope (LEXT OLS4100, Olympus, Tokyo, Japan).

### 2.8. Statistical Analysis

Statistical data analyses were conducted using a one-way analysis of variance (ANOVA) and *t* student test. The results are expressed as the mean and an Interval Confidence of 95%.

## 3. Results and Discussion

Table 2 shows the amount of G20CaII glass used as filler, the firing temperature, the value of the crystallinity index (*CI* %), and the crystalline phases identified by XRD for each sample. The firing temperatures (900 and 1100 °C) were adopted based on a previous work on WD glass-ceramics [26].

We can observe from Table 2 that all samples yielded mainly diopside (CaMgSi_2_O_6_) and wollastonite (CaSiO_3_). The crystallization of these phases was favored by the addition of G20CaII glass as a filler and a higher firing temperature. Combeite (Na_2_Ca_2_Si_3_O_9_) was only detected in samples fired at 900 °C with a 70 wt %. (WD3-900) and 100 wt %. (WD4-900) of G20CaII. Minor traces of akermanite (Ca_2_MgSi_2_O_7_), quartz (SiO_2_) and other silicates were identified. The *CI*% of the glass-ceramics heat treated up to 900 °C increased from 58% to 68%, as the amount of G20CaII increased. On the contrary, the *CI*% decreased with the glass content at 1100 °C (WD1-1100 presents a *CI*% of 73%, while WD2-1100 shows *CI*% of 55%).

In a previous study, Fiocco et al. [26] described how the introduction of G20CaII particles as a secondary filler permits the formation of very well-defined wollastonite and diopside peaks, even at 900 °C. The lower *CI*% of the WD2-1100 sample compared to WD1-1100 could be justified by the dissolution of the crystals within the surrounding softened glass phase.

**Figure 1:**
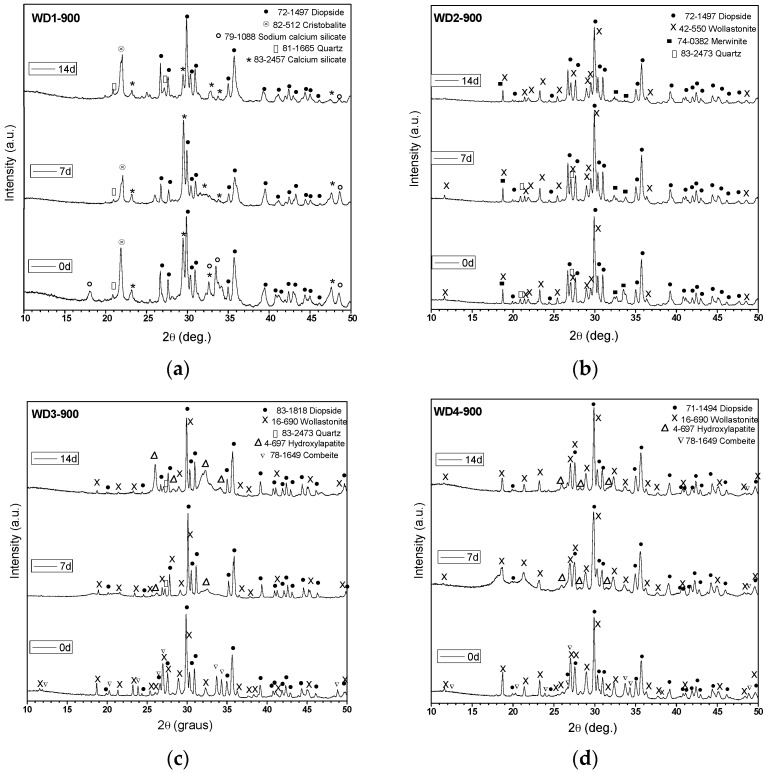

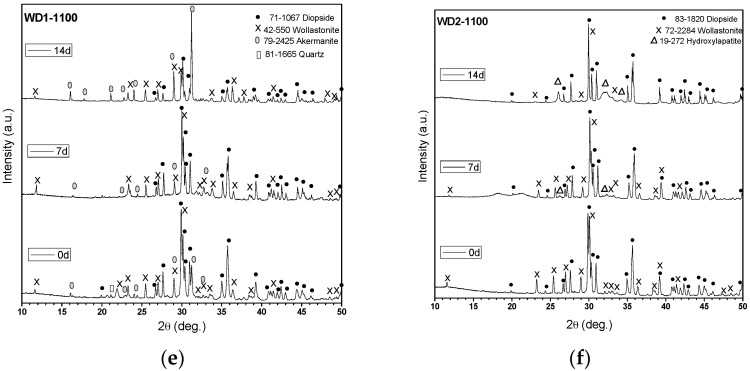
XRD patterns of polymer-derived glass-ceramics (**a**) WD1-900; (**b**) WD2-900; (**c**) WD3-900; (**d**) WD4-900; (**e**) WD1-1100; (**f**) WD2-1100, before and after soaking in SBF for seven and 14 days.

Figure 1 shows the XRD patterns of glass-ceramics before and after soaking in SBF for seven and 14 days. As can be seen, the glass-ceramics WD3-900, WD4-900, and WD2-1100 exhibited distinctive XRD peaks (2θ = 26.0°, 28.2°, 31.6°, and 34.2°) of crystalline apatite (Ca_10_(PO_4_)_6_(OH)_2_) after immersion in SBF for seven days, indicating the formation of an apatite-like layer on their surface and confirming their bioactivity. However, the broadening of the XRD peaks indicates that the crystallinity of the apatite is not high [4,6]. WD3-900 glass-ceramic shows more intense hydroxyapatite XRD peaks than WD4-900, developed from the silicone-free glass powders, and these are even more evident after the immersion in SBF for 14 days (see Figure 1c,d). As shown in Table 2, these samples present the same value of *CI*% (68%) and the same crystalline phases; the different behavior could be due to a slightly higher content of combeite in WD3-900 (considering the intensity of peaks). As pointed out by Peitl et al., combeite (Na_2_Ca_2_Si_3_O_9_) is highly bioactive [12]. Moreover, from the data in Figure 1c,d, it is apparent that the diffraction peaks of the combeite phase disappeared from the XRD spectrum after soaking in SBF for seven days, leaving the diffraction peaks of the HA phase. These findings are in agreement with those of Chen et al. [35], who showed the transformation of Na_2_Ca_2_Si_3_O_9_ to the amorphous calcium phosphate and, therefore, the bioactivity and degradability of the material.

The WD2-1100 glass-ceramic, which presents 45% of amorphous phase, and only diopside and wollastonite as crystalline phases (Figure 1f), also exhibits surface reactivity. The bioactivity of WD2-1100 might be based in the well-known bone-bonding mechanisms of bioactive glasses, as originally proposed by Hench and Wilson [36]. By contrast, the formation of HA was not observed on the surface of the WD1-900, WD2-900, and WD1-1100 samples, as shown in Figure 1a,b,e.

**Figure 2:**
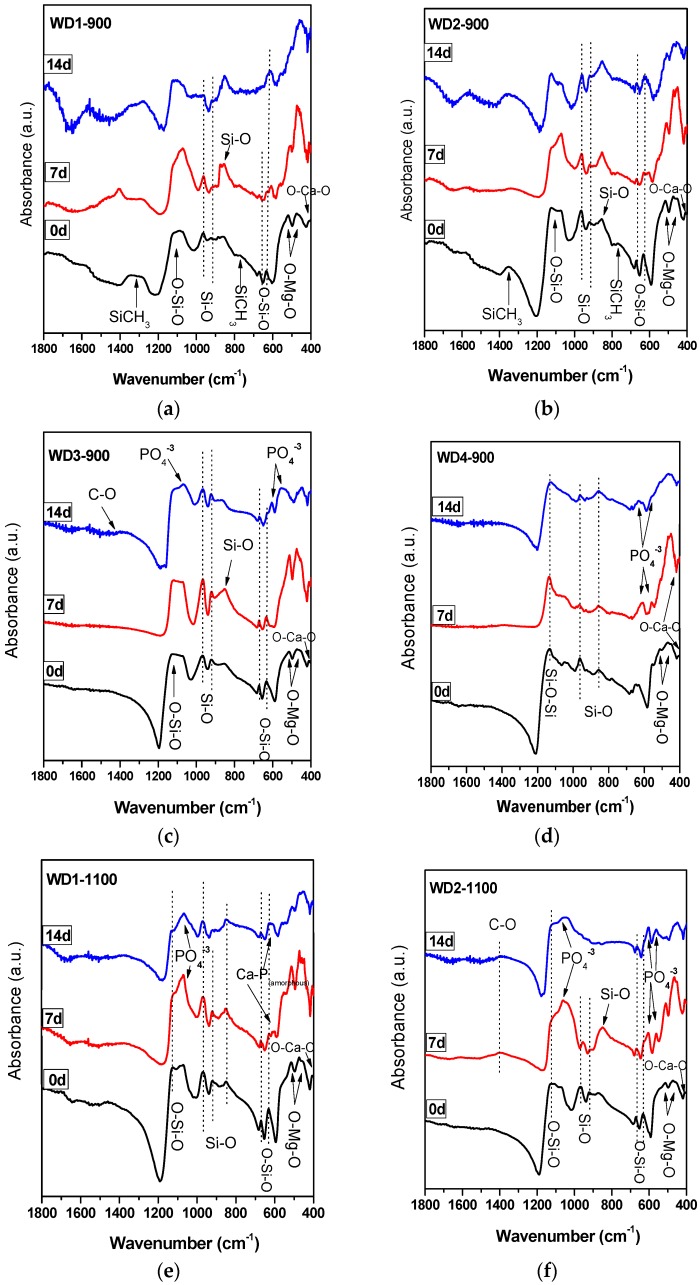
FT-IR spectra of polymer-derived glass-ceramics (**a**) WD1-900; (**b**) WD2-900; (**c**) WD3-900; (**d**) WD4-900; (**e**) WD1-1100; (**f**) WD2-1100, before and after soaking in SBF for seven and 14 days.

The FT-IR spectra of the WD glass-ceramics before and after immersion in SBF solution for seven and 14 days are presented in Figure 2. From Figure 2a,b, it is apparent that no significant differences were found between the FT-IR spectra along the immersion time for the samples WD1-900 and WD2-900. The most striking result emerging from the data is that the band consistent with the presence of residual Si–CH_3_ bonds can be noted in the spectra of samples after firing, but decreased after the immersion in SBF solution. The bands attributed to the Si–CH_3_ bonds are 1274 cm^−1^ sym. def. and 771, 697 cm^−1^ [17].

In addition, it can be seen from Figure 2c,d,f that WD3-900, WD4-900, and WD2-1100 reported a significantly increased apatite forming ability than the other samples. The gradual formation of the peaks at 1100 and 550–600 cm^−1^ after soaking in SBF indicates the formation of a Ca–P layer on the surface of the samples. The bands at 1100 cm^−1^ and 1033 cm^−1^ are attributed to the P–O stretching vibration modes, and the bands at 605 cm^−1^ and 565 cm^−1^ are attributed to the O–P–O bending mode. This is the most characteristic region for apatite and other phosphates, as it corresponds to P–O bending vibrations in a PO_4_^3−^ tetrahedron. Further, the band near 1400 cm^−1^ present in WD3-900 and WD2-1100 corresponds to the incorporation of carbonate into the apatite, resulting in a hydroxycarbonate apatite. The broad CO_3_^2−^ band detected at 1440 cm^−1^ observed after the immersion in SBF indicates A-type substitution (i.e., carbonate replacing a hydroxyl group) and the CO_3_^2−^-signal is thus shifted to lower wave numbers, starting from 1410 cm^−1^, for B-type substitution (i.e., carbonate replacing phosphate group).

A single band at 565 cm^−1^, as shown in Figure 2e, suggests the presence of non-apatitic or amorphous calcium phosphate in WD1-1100, which is usually considered as an indication of the presence of HA precursors. However, apart from a band at 565 cm^−1^, no other bands could be observed for WD1-1100, even after 14 days of immersion in the SBF solution. Besides that, WD1-1100 glass-ceramic shows an increase in the band at 1100 cm^−1^ (attributed to the P–O) due to the increasing immersion time in the SBF. Nevertheless, the XRD data of the same sample, as presented in Figure 1e, does not reveal the presence of a peak corresponding to the formation of HA.

**Figure 3:**
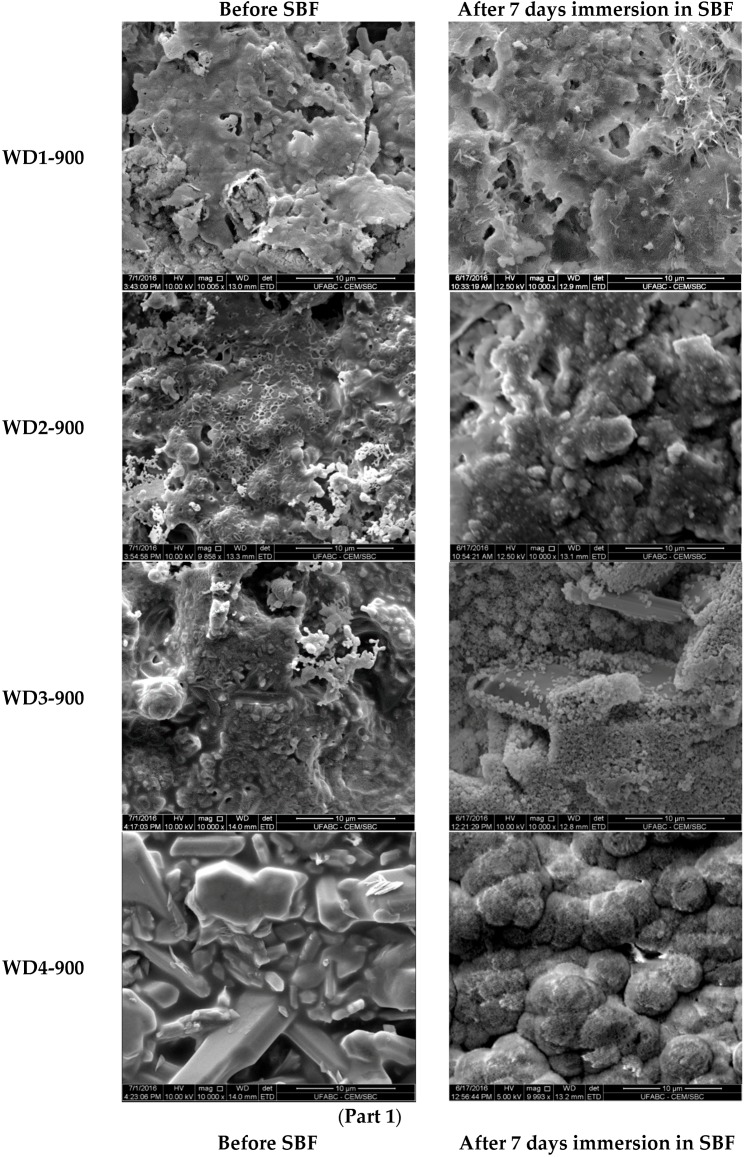

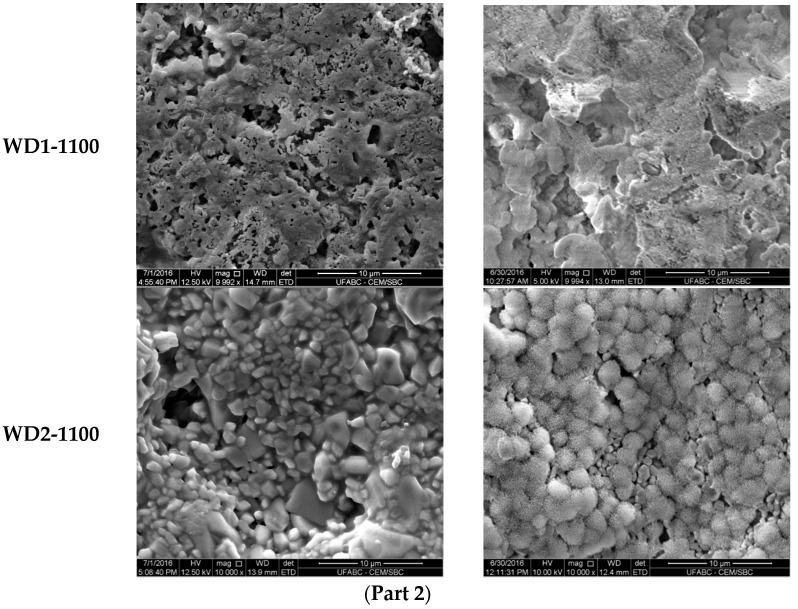
(**Part 1**). SEM surfaces micrographs of WD glass-ceramics samples before (**left**) and after (**right**) soaking in SBF solution for seven days; (**Part 2**). SEM surfaces micrographs of WD glass-ceramics samples before (**left**) and after (**right**) soaking in SBF solution for seven days.

The SEM micrographs of the surfaces for various samples before and after soaking in SBF for seven days are shown in Figure 3. The images on the left indicate that the surface morphology of the WD glass-ceramics before immersion in SBF depends on the crystalline phase composition and the firing temperature. WD1-900 (glass-free) presents a small amount of agglomerated particles and pores under 10 μm. The addition of glass as a filler gradually reduces the porosity, as can be seen in WD2-900 and WD3-900. The WD4-900 sample, obtained only from glass powders, clearly shows crystals embedded in a compact residual glassy phase. The increase in the firing temperature improves the densification of WD1-1100 and WD2-1100, evidently closer to WD4-900, despite being mostly silicone-based; a high content of crystals under 5 μm is observed in the WD2-1100 surface. The role of G20CaII addition on the densification of WD glass-ceramics has already been discussed in previous work, for a different polymer [26]; the present results provide a valuable confirmation. On the basis of XRD analysis and SEM observations, we can say that the thermal treatment of a silicone, combined with fillers, acting both as CaO and MgO precursors and “providers of liquid phase” (Na phosphate), actually leads to a wollastonite-diopside glass-ceramic close to a “standard” wollastonite-diopside glass-ceramic, from the evolution of G20CaII glass (WD4 series), but only in specific conditions. Polymer-based formulations can be an alternative to pure G20CaII glass by a temperature increase (from 900 to 1100 °C) and with at least a limited addition of the same glass.

Two important properties of cell-biomaterial interaction are the surface chemistry/energy and morphology [37]. Some studies have aimed to modify the surface to enhance the hydrophilicity, and consequently, the bioactivity, and cellular attachment and proliferation [38,39,40]. On the other hand, a designed topography is crucial to promote successful osseointegration. As mentioned in the literature review, rough surfaces exhibit a better bone response than those which are smooth [41,42]. Moreover, the combination of micro- and nano-scale roughness could improve osteoblast differentiation and influence the development of the osteoblast phenotype required to regulate the cell life cycle [37,43]. Albrektsson and Wennerberg [44] suggested that an ideal topography to induce the strongest bone response may present a moderate roughness (1–2 μm).

The increase of the surface density and presence of pores with a diameter of less than 5 μm on WD2-1100 suggests that these samples would present the appropriate surface for osteoblast adhesion and proliferation, as the material combines the physical and chemical properties expected for an active bonding with bone tissue.

Regarding the question of bioactivity, after seven days of immersion in SBF, the SEM microphotographs (Figure 3, right side) revealed: (1) no significant differences were found on the surface of WD1-900, WD2-900, and WD1-1100; and (2) strong evidence of HCA’s formation ability in SBF of WD3-900, WD4-900, and WD2-1100.

Glass-rich formulations, fired at 900 °C, were likely eroded, in SBF, as an effect of the dissolution of the combeite grains in the SBF during the period of immersion, forming a porous structure layer parallel to the SBF–sample interface. It is interesting to note, whereas spherical clusters—with a diameter of about 10 μm—formed in the WD4-900 sample, small spherical clusters with a diameter of about 2 μm (covering the whole surface of the material) formed in WD2-1100, featuring no combeite. With the support of analogous results in the literature [4,8,10], we can posit that WD3-900, WD4-900, and WD2-1100 exhibit a good rate of in vitro bioactivity.

**Figure 4:**
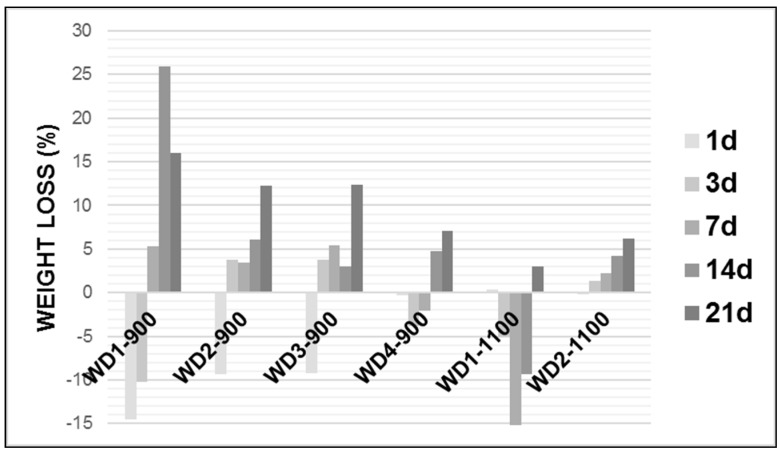
Weight loss (%) of WD glass-ceramic samples after soaking in Tris-HCl solution for 1, 3, 7, 14, and 21 days.

In order to study in vitro degradation, the wollastonite–diopside glass-ceramics were soaked in Tris-HCl solution at pH 7.4, 37.0 ± 1 °C for 1, 3, 7, 14, and 21 days. Figure 4 shows the weight loss of the glass-ceramic samples after soaking in the Tris-HCl solution.

As can be seen in Figure 4, in general, the weight loss of each glass-ceramic increased with an increasing soaking time and the degradation rate of the early period was lower than that of latter. Except for WD2-1100, all WD glass-ceramics showed a significant mass gain in the early periods, followed by a further weight loss after up to 21 days of immersion. The WD1-900 sample presented the highest value of the degradation rate after 14 days soaking in Tris-HCl solution, whereas WD2-1100 presents the lowest value for the same period of time, reaching around a 25% and 4% weight loss, respectively. For the WD2-1100 sample, there was a gradual weight loss during the observed time, with a value of up to 6%.

The degradation rate of the materials can be associated with the amount of amorphous phase. In fact, the decreasing trend of the degradation rate, for the materials fired at 900 °C, passing from WD1-900 (no glass) to WD4-900 (only glass), is parallel to that of the amount of amorphous phase, passing from 42% to 32% (see Table 2). For the materials fired at 1100 °C, the correlation is confirmed: WD2-1100 presents a higher degradation rate than WD1-1100, in agreement with the enhanced amorphous phase content (45% against 27%, see Table 2). It should be noted, however, that these data must be interpreted with caution, because in vitro tests on the degradation and surface precipitation of HA occur simultaneously on materials that can be considered bioactive [45,46]. For instance, WD4-900 and WD2-1100 present bioactivity and weight loss values under 7%, after up to 21 days of immersion in the Tris-HCl solution. More studies are needed to explain a mass gain in early periods.

**Figure 5:**
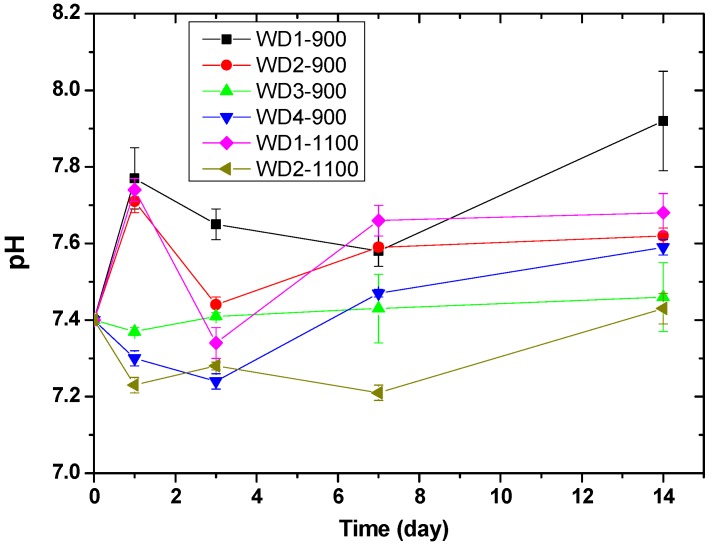
pH of Tris-HCl solution as a function of the immersion time of WD glass-ceramics. The Tris-HCl solution was replaced after every measurement.

Figure 5 shows the pH value of the Tris-HCl solution as a function of the immersion time of WD glass-ceramics. The change in pH values can be related to the exchange rate of ions. The pH values of WD1-900, WD2-900, and WD1-1100 rise from 7.4 to 7.8 on the first day of immersion, probably due to the rapid ion exchange between Ca^2+^ and Na^+^ in the surface of the samples and H^+^ or H_3_O^+^ in the Tris-HCl solution [47,48]. After three days of immersion, a decrease in pH from 7.8 to 7.4 was observed for the WD2-900 and WD1-1100 samples. After this time, the pH value increased gradually until 14 days. Conversely, for WD3-900, WD4-900, and WD2-1100, no significant differences were observed in the pH values during the immersion time, and just a slight decrease in pH from 7.4 to 7.2 was observed for WD2-1100 on day one of the immersion. For immersion times longer than one day, the pH value slowly increases to approximately 7.4 in 14 days.

These findings are in agreement with Kaur et al. [49], who suggested that the change in weight is due to the exchange of ions between the surface of the sample and the solution. This ion exchange leads to a change in the pH value of the solution, which further causes the formation of new stable phases. A higher weight loss leads to a rapid change in the pH with the increase in the concentration of ions in the solution.

**Figure 6:**
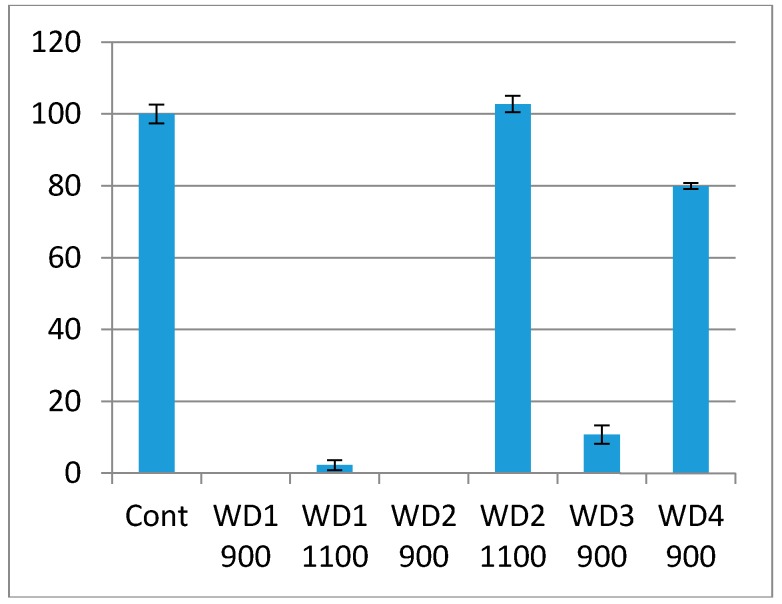
Cell viability of Balb/c cells when exposed to the extract samples. The viability is expressed as the percentage of viable cell compared to the control, and the bars are the confidential intervals when α = 0.05.

The cytotoxicity test using the indirect method is based on an extract of the samples. The samples were immersed in culture medium for 72 h at 37 °C under gentle shaking. The culture medium in which the samples were soaked was used in the test. The culture medium had a pH indicator, phenol red, which changes the color from red to pink when there are substances which increase the pH, or red to yellow when there are substances which decrease the pH. As shown in Figure 6, the WD1-900, WD1-1100, WD2-900, and WD3-900 samples reacted with the culture medium, turning them pink, and the extract killed most of the cells in this test. This corroborates Figure 5, in which the changes of the pH value are shown and the resulting ions were discussed. It can also be seen from Figure 6 that the WD4-900 and WD2-1100 samples are considered non cytotoxic according to the ISO10993-5, which says that the material can be considered cytotoxic when 30% of the cell viability is lost.

A remark should be dedicated to the lithium content. As shown in Table 2 and Figure 1, there was no crystal phase including lithium, so that Li^+^ ions remained in the residual glass phase. The ionic concentration of Li^+^ ions, presented in G20CaII, is lower than the minimum values required for therapeutic or toxic effects to occur. In low amounts, the presence of Li^+^ ions can enhance the bone formation trough intracellular pathways [50], and increase bone density [51]. The decrease of G20CaII, passing from WD4-900 (only glass) to WD2-1100, evidently reduces the availability of Li^+^ ions.

**Figure 7:**
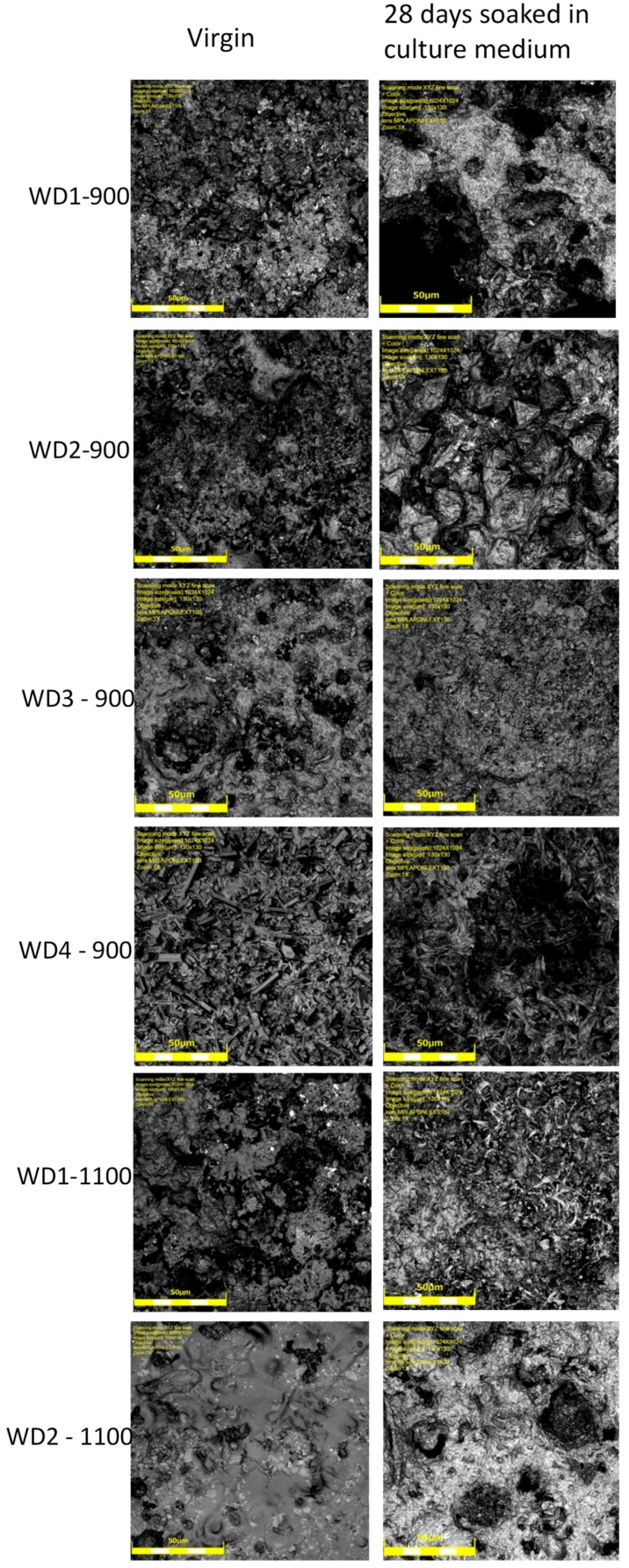
Surface characterization by Laser Confocal Microscopy (Olympus LEXT OLS4100). Objective: 100×.

The degradation of the samples can also be observed by a comparison of the laser confocal microscopy images, shown in Figure 7. In general, all of the samples changed their surface after having been soaked in the culture medium for 28 days. In particular, we can observe an increase in the porosity and smoothness of the surfaces. The smoothness can be explained by the accumulation of calcium on the surface of the samples; this observation can be confirmed by dying with Alizarin red, as it will be shown later.

**Figure 8:**
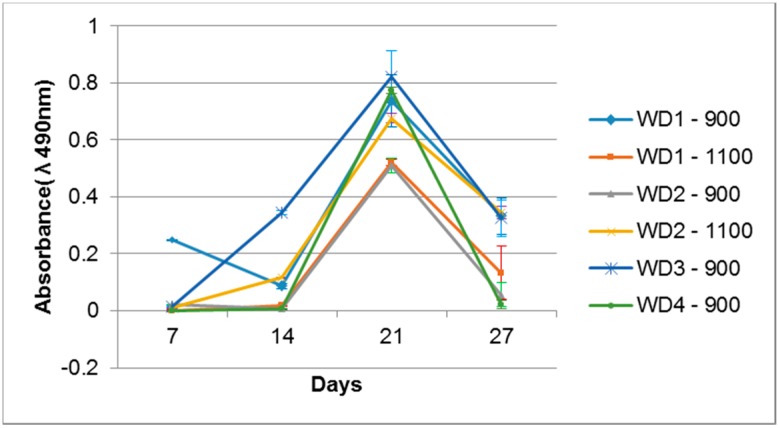
MG63 cell activity measured by MTS when seeded onto the WD glass-ceramic surfaces.

Cell activity on the sample surfaces was observed by MTS, where the proportion sample surface/volume was kept constant. The optical density of the samples is shown in Figure 8. All the samples were evaluated with their own blank sample, to be sure that there is no reactive compound released which could change the MTS color. After seven days of cell cultivation, only the WD1-900 sample showed a significant color intensity, which decreased after 14 days and rose after 21 days. The WD2-1100 and WD3-900 samples had a detectable color formation at 14 days, and were linear after 21 days. After 21 days of cell cultivation, all samples presented the formation of color, which decreased drastically after 28 days of culture. This phenomenon could be correlated to the surface changes observed in Figure 7, and the degradation test in Figure 4. The cells probably detached from the sample after 27 days of culture.

**Figure 9:**
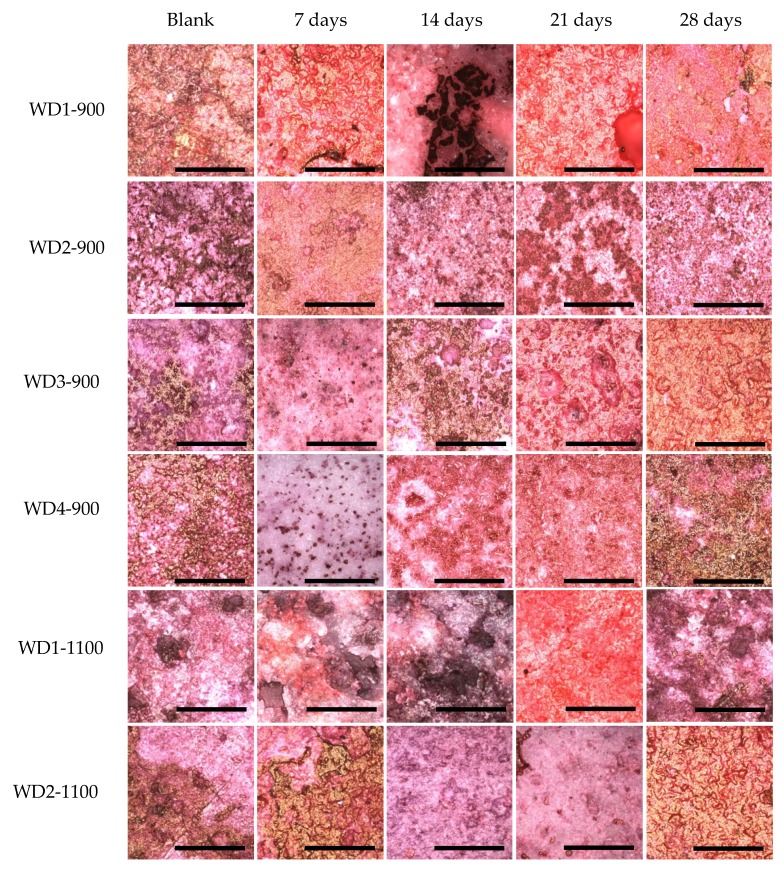
Alizarin Red S staining of the WD glass-ceramic samples. The blank sample was immersed in culture medium for 28 days. For the other samples, 2 × 10^4^ cells were seeded on the surface of the samples and cultivated for 7, 14, 21, and 28 days. Objective: 20×. Scale bar = 200 μm.

The calcification was observed by staining the samples with alizarin Red S. This compound is widely used to stain calcium. Alizarin Red S complexes with the calcium, and a red color is formed. The association of the calcium with other compounds can vary the color to yellow or brown, especially when it is bound to phosphate, as in the case of hydroxyapatite in the bone mineral structure [52]. Figure 9 shows these differences.

For all samples soaked in the culture medium, calcium bonding on the surface can be detected, with the red background. The samples also showed a yellowish-brownish color, which indicates a bone-like structure. In order to achieve a bone-like structure, it is important that the material presents clusters for the nucleation of calcium phosphates. When the cells were seeded on the samples, a homogeneity of the staining was observed. This phenomenon occurs by the interaction of the cells, which start to produce collagen as extracellular matrix, and this collagen gives direction to the calcium, resulting in phosphate nucleation [53,54,55,56].

After 28 days of culturing, the most significant change in color was observed for the WD4-900 sample, followed by WD2-1100 and WD3-900. In Figure 9, however, the WD2-1100 sample shows a more homogenous staining. Using the results of the cytotoxicity test (Figure 6), the WD2-1100 sample can be considered as the best. Moreover, we can observe that the blank for itself has a degree of bone-like staining, corroborating the results of bioactivity with the SBF solution. For WD2-1100, two phenomena occur at the same time: cell proliferation and calcium deposition. The combination of silicone, reactive fillers, and G20CaII, for firing at 1100 °C, yields a material that follows the original theory of the bone-bonding mechanism proposed by Hench and Wilson [36].

## 4. Conclusions

This paper has described an extensive evaluation of the biological behavior of wollastonite–diopside glass-ceramics, from a non-conventional route, i.e., based on a silicone polymer and fillers, including G20CaII glass particles. It was found that bioactivity depends on the crystalline phases formed in the glass-ceramics. Glass-ceramics which presented wollastonite, diopside, and combeite (WD3-900 and WD4-900), or only wollastonite and diopside (WD2-1100), showed good bioactivity. The addition of G20CaII particles as a filler favors the formation of these phases and therefore improves the bioactivity. The degradation rate of the WD glass-ceramics is mainly dependent on the crystalline fraction: the higher the crystalline fraction, the lower the rate of the degradation of glass-ceramics fired at 900 or 1100 °C. The WD2-1100 glass-ceramic samples showed good bioactivity and no cytotoxicity, and the evidence from this research suggests that this sample may be successfully used as a bone defect filler in the Tissue Engineering area. The results of this specific sample also undoubtedly support the current studies on the 3D printing of wollastonite-diopside glass-ceramics by using similar compositions. Further work needs to be done to establish whether the best WD2-1100 candidate also demonstrates biocompatibility, using in vivo tests.

## Figures and Tables

**Table 1:** Chemical composition of the G20CaII glass.

Glass Composition (% mol)	SiO_2_	CaO	MgO	Na_2_O	Li_2_O
G20CaII	55.3	22.0	12.0	9.0	1.7

**Table 2:** Crystalline phases identified in polymer-derived glass-ceramics by XRD analysis.

Sample	Glass Content (wt %)	Firing T (°C)	Crystallinity Index (*CI* %)	Crystalline Phases (PDF#)
WD1-900	0	900	58	Diopside (72-1497), Wollastonite (83-2457), Sodium Calcium silicate (79-1088), Cristobalite low (82-512), Quartz (81-1665)
WD2-900	30	900	65	Diopside (75-1497), Wollastonite (42-550), Merwinite (74-0382), quartz (83-2473)
WD3-900	70	900	68	Diopside (83-1818), Wollastonite (16-690), Combeite (78-1649)
WD4-900	100	900	68	Diopside (71-1494), Wollastonite (16-690), Combeite (78-1649)
WD1-1100	0	1100	73	Diopside (71-1067), Wollastonite (42-550), Akermanite (79-2425), Quartz (81-1665)
WD2-1100	30	1100	55	Diopside (83-1818), Wollastonite (72-2284)
